# Three-Dimensional MRI-Based Statistical Shape Analysis of Knee Bone Morphology in Individuals with Generalized Joint Hypermobility

**DOI:** 10.3390/bioengineering13101202

**Published:** 2026-10-15

**Authors:** Ye Luo, Luqi Guo, Zhitao Zhu, Feng Li, Xinwei Huang, Shaobai Wang, Yuanbiao Luo

**Affiliations:** 1 Department of Orthopaedics, The First Hospital of Putian City, The School of Clinical Medicine, Fujian Medical University, Putian 351100, China; 2 School of Exercise and Health, Shanghai University of Sport, Shanghai 200438, China; 3 Department of Rehabilitation Therapy, Yangzhi Affiliated Rehabilitation Hospital of Tongji University, Shanghai 201619, China

**Keywords:** generalized joint hypermobility, statistical shape modeling, knee bone morphology, magnetic resonance imaging, principal component analysis, three-dimensional bone morphology

## Abstract

Background: Generalized joint hypermobility (GJH) is associated with ligamentous and capsular laxity and an increased risk of knee injury. Whether GJH is associated with three-dimensional knee bone morphology remains unclear. This study compared femoral, tibial, and patellar morphology between young adults with GJH and healthy controls using magnetic resonance imaging (MRI)-based statistical shape modeling (SSM). Methods: This cross-sectional study included 80 participants aged 18–25 years, comprising 40 individuals with GJH (Beighton score ≥ 6) and 40 healthy controls (Beighton score ≤ 2). One right knee per participant was analyzed. High-resolution 3.0-T MRI was used to segment and model the femur, tibia, and patella separately in ShapeWorks. The primary SSMs were scale-normalized using Procrustes alignment to remove global isotropic scale differences. Principal components (PCs) accounting for at least 95% of cumulative shape variance were retained. Overall group differences were assessed using permutational multivariate analysis of variance (PERMANOVA), with multivariate dispersion assessed using PERMDISP. Individual PCs were compared with Benjamini–Hochberg false discovery rate (FDR) correction. Results: Significant overall scale-normalized shape differences were observed between the GJH and healthy control groups for the femur (pseudo-F = 8.14, R^2^ = 0.0945, *p* = 0.0009), tibia (pseudo-F = 9.13, R^2^ = 0.1048, *p* = 0.0006), and patella (pseudo-F = 2.00, R^2^ = 0.0250, *p* = 0.0255), with no significant between-group differences in multivariate dispersion. After FDR correction, femoral PC1 (Hedges’ g = 0.765, q = 0.0171) and tibial PC1 (g = 0.841, q = 0.00621) differed significantly between groups, whereas no individual patellar PC remained significant. Descriptive surface maps showed visually larger group-mean surface displacements in periarticular osseous contours than across the major articular contact surfaces. Conclusions: Significant three-dimensional differences in femoral, tibial, and patellar morphology remained detectable between individuals with GJH and healthy controls after removal of global isotropic scale. Femoral and tibial differences were primarily reflected in PC1, whereas the patella showed an overall multivariate difference without an individual FDR-significant PC. After global isotropic scale was removed, between-group shape differences persisted, indicating that they were not solely attributable to overall bone size.

## 1. Introduction

Generalized joint hypermobility (GJH) refers to a range of motion exceeding normal physiological limits across multiple joints and is commonly associated with laxity of the ligaments, joint capsules, and other connective tissues [1,2,3]. GJH is relatively common in young and athletic populations. Most individuals with GJH are asymptomatic, but some experience joint instability, recurrent sprains, pain, fatigue, and difficulties with motor control [1,2,3]. GJH has been proposed to involve alterations in passive stabilizing structures, active muscular control, and proprioceptive feedback [4]. Therefore, GJH is characterized not only by increased joint mobility but also by reduced joint stability, impaired motor control, and an increased risk of injury.

The knee is an important site of sport-related injury associated with GJH. Knee stability depends on the coordinated contribution of osseous structures, ligaments and joint capsules, and muscular control. In individuals with GJH, ligamentous and capsular laxity may reduce passive stability, making the knee more susceptible to inadequate control during terminal extension, hyperextension, landing, cutting, and single-leg support. Previous systematic reviews have shown that GJH is associated with an increased risk of lower-limb joint injury during sports, with knee injury being of particular concern [5]. Another systematic review reported that GJH is associated not only with an increased risk of anterior cruciate ligament (ACL) injury but also with poorer outcomes after ACL reconstruction [6]. In addition to the tibiofemoral joint, the patellofemoral joint may also be affected by GJH. A recent systematic review identified an association between joint hypermobility and patellar instability, suggesting that GJH may be an important risk factor for patellar dislocation or instability [7].

Current explanations for the increased risk of knee injury in individuals with GJH have focused mainly on soft-tissue laxity and neuromuscular control. Reduced proprioceptive acuity has been reported in children with joint hypermobility when the knee moves into the hypermobile range, suggesting that individuals with GJH may have impaired position sense and control near the end range of motion [8]. Clinically, individuals with GJH may rely more heavily on active muscular control to maintain joint stability, particularly when ligamentous restraint is insufficient, the joint approaches its terminal position, or external perturbations occur. However, knee stability is also influenced by osseous structure. The femoral condyles, tibial plateau, intercondylar eminence, trochlear morphology, and patellar contour together form the osseous boundaries and motion-guiding structures of the knee and can influence tibiofemoral congruence, rotational control, patellar tracking, and patellofemoral stability.

Conventional imaging studies of osseous structure commonly use individual angular, distance, or ratio-based measurements to describe bone morphology, including posterior tibial slope, trochlear groove depth, tibial tubercle–trochlear groove distance, patellar height, and patellar tilt [9,10,11]. These measures have clear clinical value but cannot fully characterize complex three-dimensional bone morphology. Previous MRI-based research has also identified differences in several patellofemoral morphological parameters between individuals with global joint hypermobility and controls, including lateral trochlear inclination, trochlear facet asymmetry, trochlear groove depth, patellar lateralization, sulcus angle, and central trochlear height [12]. However, these analyses relied on predefined regional measurements and did not comprehensively characterize three-dimensional bone morphology.

Statistical shape modeling (SSM) reconstructs bone surfaces from three-dimensional imaging and extracts the major modes of morphological variation within a population, allowing for comprehensive three-dimensional assessment of shape differences. In recent years, SSM has been applied to ACL injury, patellofemoral instability, knee osteoarthritis, and knee biomechanics, demonstrating its potential to reveal relationships between bone morphology, joint function, and disease risk [13,14,15]. However, these studies have not jointly modeled the femur, tibia, and patella in individuals with GJH. To address this gap, we used 3D MRI and SSM to compare femoral, tibial, and patellar morphology between individuals with GJH and healthy controls. We hypothesized that individuals with GJH would exhibit detectable three-dimensional shape differences in these bones.

## 2. Materials and Methods

### 2.1. Participants

This cross-sectional three-dimensional imaging study was designed to compare knee bone morphology between individuals with generalized joint hypermobility and healthy controls using knee MRI and SSM. Participants were recruited through public recruitment. The study was approved by the Ethics Committee of Shanghai University of Sport (No. 102772025RT171) and was conducted in accordance with the Declaration of Helsinki. Before participation, all participants were informed of the study purpose, imaging procedures, and potential risks and provided written informed consent.

Because directly comparable MRI-based SSM data were unavailable, sample adequacy was evaluated using an approximate conventional two-group calculation. A previous MRI-based study comparing knee morphology between individuals with global joint hypermobility and healthy controls included 42 individuals per group [12]. A standardized effect size of Cohen’s d = 0.65 was therefore assumed as an approximate reference for sample adequacy rather than as an analysis-specific power calculation for the multivariate SSM/PERMANOVA framework. Assuming a two-sided significance level of 0.05, a statistical power of 80%, and an equal allocation ratio between the GJH and HC groups, a minimum of 39 individuals per group was required. The final analytical sample included 40 individuals in each group, which exceeded the estimated minimum and was comparable to the previous MRI-based study [12].

GJH was assessed using the Beighton score [3]. The Beighton score is the most widely used nine-point assessment for generalized joint hypermobility and includes bilateral fifth-finger dorsiflexion, thumb-to-forearm apposition, elbow hyperextension, knee hyperextension, and forward trunk flexion with the palms placed on the floor [16]. Participants were assigned to the GJH group or healthy control (HC) group according to their Beighton score.

The inclusion criteria were as follows: (1) age 18–25 years; (2) eligibility for MRI examination without relevant contraindications; (3) no history of lower-limb injury; and (4) a Beighton score ≥ 6 for the GJH group or ≤2 for the HC group [16,17].

The exclusion criteria were as follows: (1) previous knee surgery; (2) acute lower-limb injury within the previous 6 months; (3) previous knee fracture or obvious osseous deformity; (4) inflammatory joint disease; (5) systemic disease affecting bone morphology or bone metabolism; (6) active rheumatic disease; (7) neuromuscular disease; and (8) pregnancy.

### 2.2. MRI Acquisition, Image Processing, and Bone Segmentation

All participants underwent knee MRI using a 3.0-T MRI scanner (MAGNETOM Prisma, Siemens Healthcare, Erlangen, Germany). Participants were positioned supine with the knee placed in a dedicated coil, maintained in slight flexion, and stabilized to minimize motion. High-resolution three-dimensional dual-echo steady-state (DESS) imaging was used for bone reconstruction with the following parameters [18]: echo time (TE) = 5.4 ms, repetition time (TR) = 11.7 ms, slice thickness = 0.6 mm, field of view (FOV) = 160 × 160 mm, and matrix = 320 × 320. Motion artifacts were minimized during acquisition. After image acquisition, the femur, tibia, and patella were manually segmented from the three-dimensional DESS images using 3D Slicer version 5.8.1. The segmentation boundaries were reviewed in multiplanar views and three-dimensional reconstructions before the final binary labels were exported for subsequent processing.

After segmentation, all labels underwent slice-by-slice and three-dimensional quality control. Quality control included assessment of structural completeness and boundary continuity and identification of obvious under-segmentation, over-segmentation, label discontinuities, erroneous connections, or isolated small regions. Errors were manually corrected. The largest connected component of each target bone was then retained, and isolated regions unrelated to the target bone were removed. The final femoral, tibial, and patellar labels were saved as binary images for subsequent image preprocessing and statistical shape model construction.

Intra-rater segmentation reliability was assessed in 10 randomly selected right knees, each of which was independently segmented twice by the same rater. Agreement between repeated segmentations was quantified separately for the femur, tibia, and patella using the Dice similarity coefficient and average symmetric surface distance (ASSD). Three-dimensional MRI bone segmentation combined with SSM has been used to investigate knee bone morphology, ACL injury, and patellofemoral morphology and can extract three-dimensional morphological information that cannot be fully described by conventional two-dimensional measurements [13,14,15].

For statistical shape modeling, only right-knee MRI data were included to maintain consistent anatomical laterality across participants and to avoid potential effects associated with left-to-right mirroring. Participants without available right-knee MRI data were not included in the SSM analysis. Accordingly, 80 right knees from 80 participants (40 GJH and 40 HC) were used for model construction.

### 2.3. Image Preprocessing

Image preprocessing and statistical shape modeling were performed in ShapeWorks version 6.7. ShapeWorks is an open-source statistical shape modeling tool that can generate dense surface particle models with corresponding points across subjects from segmentation labels or surface models [19]. A particle represents a three-dimensional correspondence point constrained to the anatomical surface rather than a point within the bone volume. Its particle-based model uses an entropy-optimization framework to establish consistent surface correspondences across samples through group-wise optimization of particle distributions [20]. This framework balances uniform sampling of each individual surface with compactness of the population-level shape model, such that particles with the same index represent corresponding surface locations across individuals.

First, the isolate operation was used to retain the main connected component of the target bone and remove background noise and isolated small structures. The fill-holes operation was then used to fill internal holes in the labels and preserve structural continuity. Labels were subsequently cropped automatically, with a 10-voxel padding added beyond the cropped region to prevent truncation of bone margins. To reduce staircase artifacts along binary label boundaries, anti-aliasing was applied for 10 iterations. The labels were then resampled to isotropic voxels with a target voxel size of 0.6 mm. After resampling, distance-transform images were generated and Gaussian smoothing was applied with sigma = 1.5. The distance transform and smoothing converted the binary labels into implicit surface representations suitable for particle optimization and reduced the influence of local boundary noise on particle distribution.

After preprocessing, rigid registration was performed using the iterative closest point algorithm, with the reference object automatically selected by the software [21]. This step reduced non-morphological variation associated with differences in position, orientation, and pose across participants [13]. Global scale normalization for the primary SSM was subsequently performed during particle optimization.

### 2.4. Statistical Shape Model Construction and Shape Visualization

**Figure 1:**
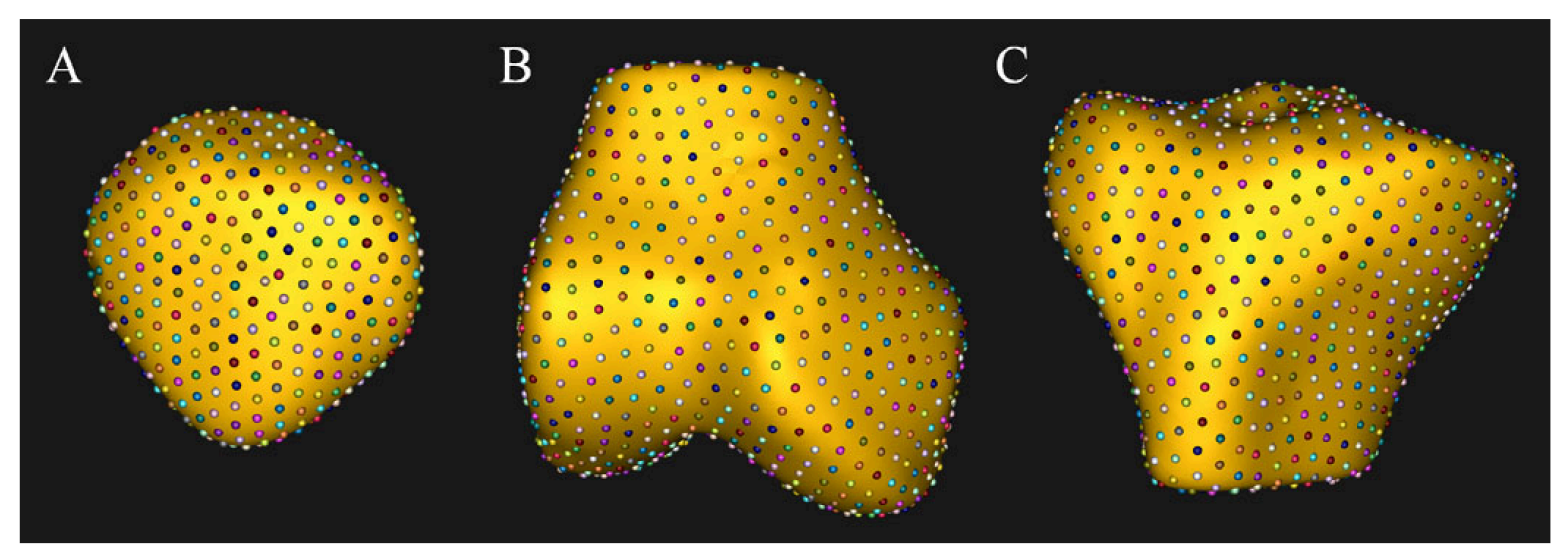
Representative surface particle distributions used for statistical shape modeling. Surface particles are shown for the (**A**) patella, (**B**) femur, and (**C**) tibia. A total of 512 particles were used for the patella and 1024 particles for the femur and tibia. Particles are constrained to the anatomical surface and jointly optimized across subjects to establish corresponding surface locations for population-level shape analysis. The three-dimensional coordinates of corresponding particles were used for principal component analysis. Particle colors are used for visualization only and do not represent anatomical regions, displacement magnitude, or statistical significance.

Three separate single-domain statistical shape models were constructed for the femur, tibia, and patella. Each bone was modeled independently to establish consistent surface correspondences across participants within the same osseous structure. Because the femur and tibia are larger and have more complex surface morphology, 1024 particles were used for each. The patella has a smaller volume and more limited surface extent, and therefore 512 particles were used. Particle numbers were selected with consideration of bone size, local morphological complexity, computational stability, and model interpretability. Representative surface particle distributions for the patellar, femoral, and tibial models are shown in Figure 1.

Particle optimization parameters were kept consistent across the three bones. The initial relative weighting was set to 0.05 and the relative weighting to 1. The regularization parameter was gradually reduced from 1000 to 10 to stabilize the covariance estimate used in the shape-space entropy calculation during the early stages of optimization and to progressively allow the population covariance structure to contribute as correspondence improved. The number of iterations per split was set to 1000, the total number of optimization iterations was 1000, and the narrow-band width was 4.

For the primary statistical shape models, Procrustes alignment with scaling, rotation, and translation was enabled during particle optimization. This procedure removed global isotropic scale differences across participants while retaining residual interindividual variation in three-dimensional shape. The same scale-normalization procedure was applied independently to the femoral, tibial, and patellar models.

After optimization, particle distributions and reconstructed surfaces were visually inspected for each model to confirm uniform coverage of the target bone surface and to identify local clustering, mismatches, or obvious surface abnormalities. Samples that failed quality control were returned to the segmentation and preprocessing steps for review. Dense surfaces for visualization were reconstructed in ShapeWorks using mesh warping, in which a reference surface mesh was deformed according to the optimized particle correspondences.

For comparison, scale-preserved models were generated using the same 80 right knees and the same anatomical inputs, particle numbers, preprocessing procedures, and particle-optimization settings as the primary models, except that isotropic scaling was not applied. These models were used specifically to assess the contribution of global bone size to the dominant PC1.

After particle optimization, a population mean shape was constructed from the corresponding particle coordinates of all samples, and principal component analysis was used to extract the major modes of morphological variation. Principal component analysis is a commonly used dimensionality-reduction method in SSM that transforms high-dimensional three-dimensional coordinate data into a set of orthogonal modes of shape variation [22]. Each principal component (PC) represents a major direction of three-dimensional morphological variation, and PC scores quantify the extent to which each individual deviates along that mode.

For each specimen, the x-, y-, and z-coordinates of all corresponding particles were concatenated into a single shape vector. PCA was then performed on these shape vectors separately for the primary scale-normalized and scale-preserved models. Thus, the principal components of the primary analysis represent residual three-dimensional shape variation after removal of global isotropic scale, whereas those of the scale-preserved models represent combined size-and-shape variation. The sign of a PC is arbitrary; positive and negative PC scores therefore represent opposite directions along the same morphological mode relative to the population mean.

### 2.5. Statistical Analysis

For each bone, PCs accounting for at least 95% of the cumulative variance in the primary scale-normalized model were retained for statistical analysis. Overall, between-group differences in three-dimensional bone shape were assessed using permutational multivariate analysis of variance (PERMANOVA) based on Euclidean distances in the retained PC space, with 9999 permutations [23]. Homogeneity of multivariate dispersion was assessed using PERMDISP with 9999 permutations. Because three primary bone-level PERMANOVA tests were conducted for the femur, tibia, and patella, family-wise error was controlled using the Holm step-down procedure. Both unadjusted and Holm-adjusted *p* values were reported. Individual PC scores were compared between groups using Welch’s independent-samples *t* test, and Hedges’ g was calculated as the effect size. To control the false-positive risk arising from multiple PC comparisons within each bone, the Benjamini–Hochberg false discovery rate (FDR) procedure was used for multiple-testing correction [24]. An FDR-adjusted q value < 0.05 was considered statistically significant.

For the scale-preserved analysis, evaluation of global bone size focused on PC1. For each bone, the variance explained by PC1 was recorded, and the association between PC1 scores and the segmented volume of the corresponding modeled region was quantified using Pearson’s correlation coefficient. Surface difference maps were generated from the displacement between corresponding locations on the GJH and HC group mean surfaces and were used to visualize the spatial distribution and direction of morphological differences. These maps were interpreted descriptively and did not represent pointwise or regional statistical significance.

## 3. Results

### 3.1. Participant Characteristics, Segmentation Reliability, and Retained Principal Components

This study included 40 participants in the GJH group and 40 participants in the HC group. Participant characteristics are presented in Table 1. There were no statistically significant between-group differences in age, sex distribution, height, weight, or body mass index (BMI) (all *p* > 0.05). The Beighton score was significantly higher in the GJH group than in the HC group (*p* < 0.001), consistent with the predefined group criteria.

Intra-rater segmentation reliability was high across all three bones. Mean Dice coefficients were 0.979 ± 0.007 for the femur, 0.979 ± 0.007 for the tibia, and 0.962 ± 0.013 for the patella. The corresponding ASSD values were 0.399 ± 0.114 mm, 0.393 ± 0.083 mm, and 0.403 ± 0.116 mm, respectively.

In the primary scale-normalized analysis, PCs accounting for at least 95% of the cumulative explained variance were retained. For the femur, PC1–PC18 were retained, accounting for 95.22% of the cumulative variance. For the tibia, PC1–PC21 were retained, accounting for 95.08%. For the patella, PC1–PC24 were retained, accounting for 95.23%. PC1 accounted for 63.64%, 62.00%, and 34.53% of the variance in the femur, tibia, and patella, respectively.

### 3.2. Overall Between-Group Differences in the Morphology of the Three Bones

Multivariate analysis based on PCs retained within the 95% cumulative-variance threshold showed significant overall scale-normalized shape differences between the GJH and HC groups for the femur, tibia, and patella. The overall femoral shape difference was significant (pseudo-F = 8.14, R^2^ = 0.0945, *p* = 0.0009, Holm-adjusted *p* = 0.0018), as was the tibial shape difference (pseudo-F = 9.13, R^2^ = 0.1048, *p* = 0.0006, Holm-adjusted *p* = 0.0018) and the patellar shape difference (pseudo-F = 2.00, R^2^ = 0.0250, *p* = 0.0255, Holm-adjusted *p* = 0.0255).

PERMDISP showed no significant between-group differences in multivariate dispersion for the femur (*p* = 0.142), tibia (*p* = 0.561), or patella (*p* = 0.791), providing no evidence that the significant PERMANOVA findings were driven by unequal within-group dispersion.

**Figure 2:**
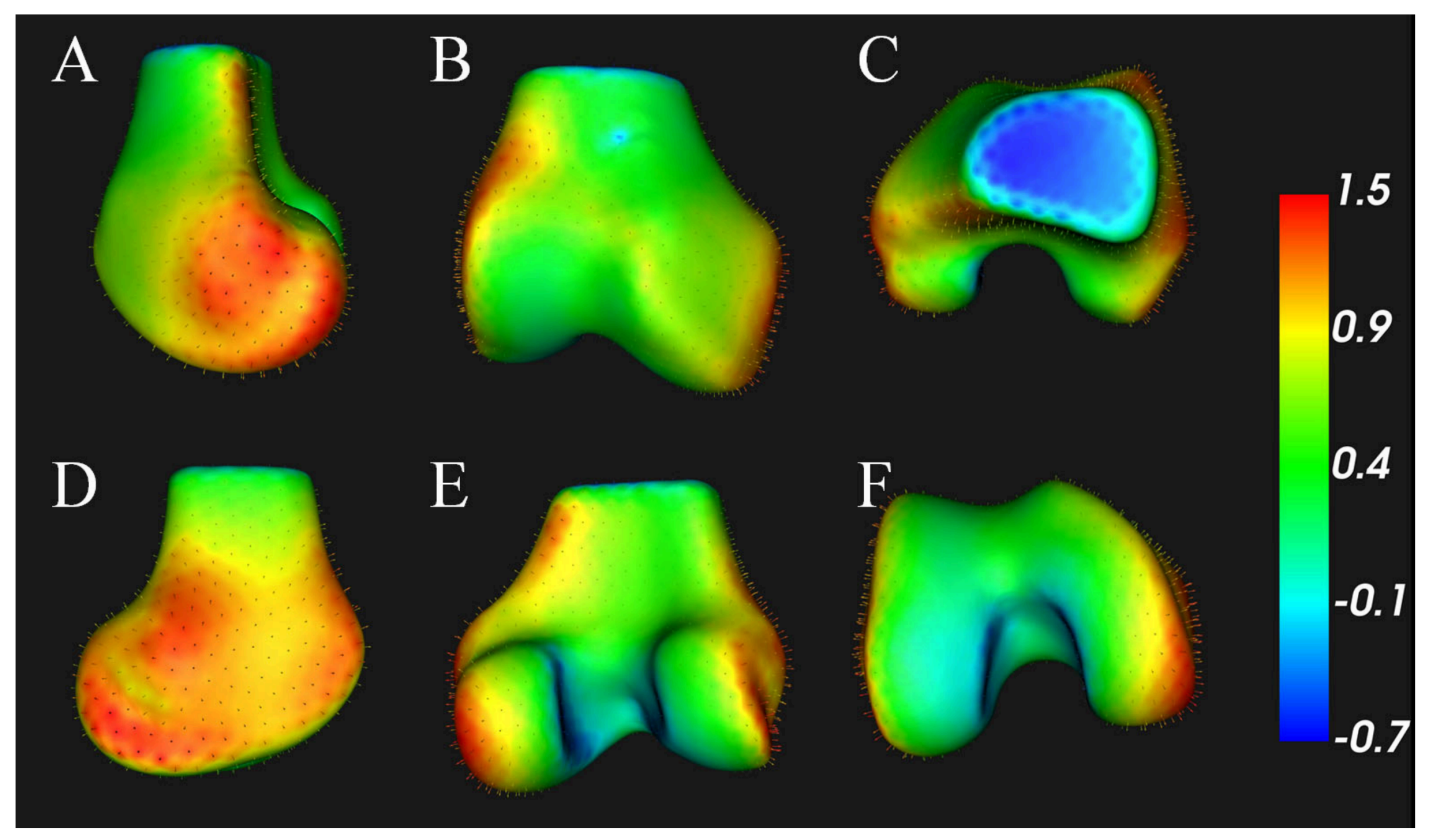
Descriptive surface map of between-group femoral shape differences. The map visualizes surface displacement between the GJH and HC group mean femoral shapes. (**A**) Left-side view; (**B**) anterior view; (**C**) superior view; (**D**) right-side view; (**E**) posterior view; (**F**) inferior view. The color bar indicates GJH−HC surface displacement (mm), with warm and cool colors representing positive and negative displacement, respectively. The more apparent displacement patterns were observed around the distal femoral periarticular contours, including the epicondylar and paracondylar regions and the lateral trochlear margin. The map is descriptive, and the colors do not indicate pointwise statistical significance.

**Figure 3:**
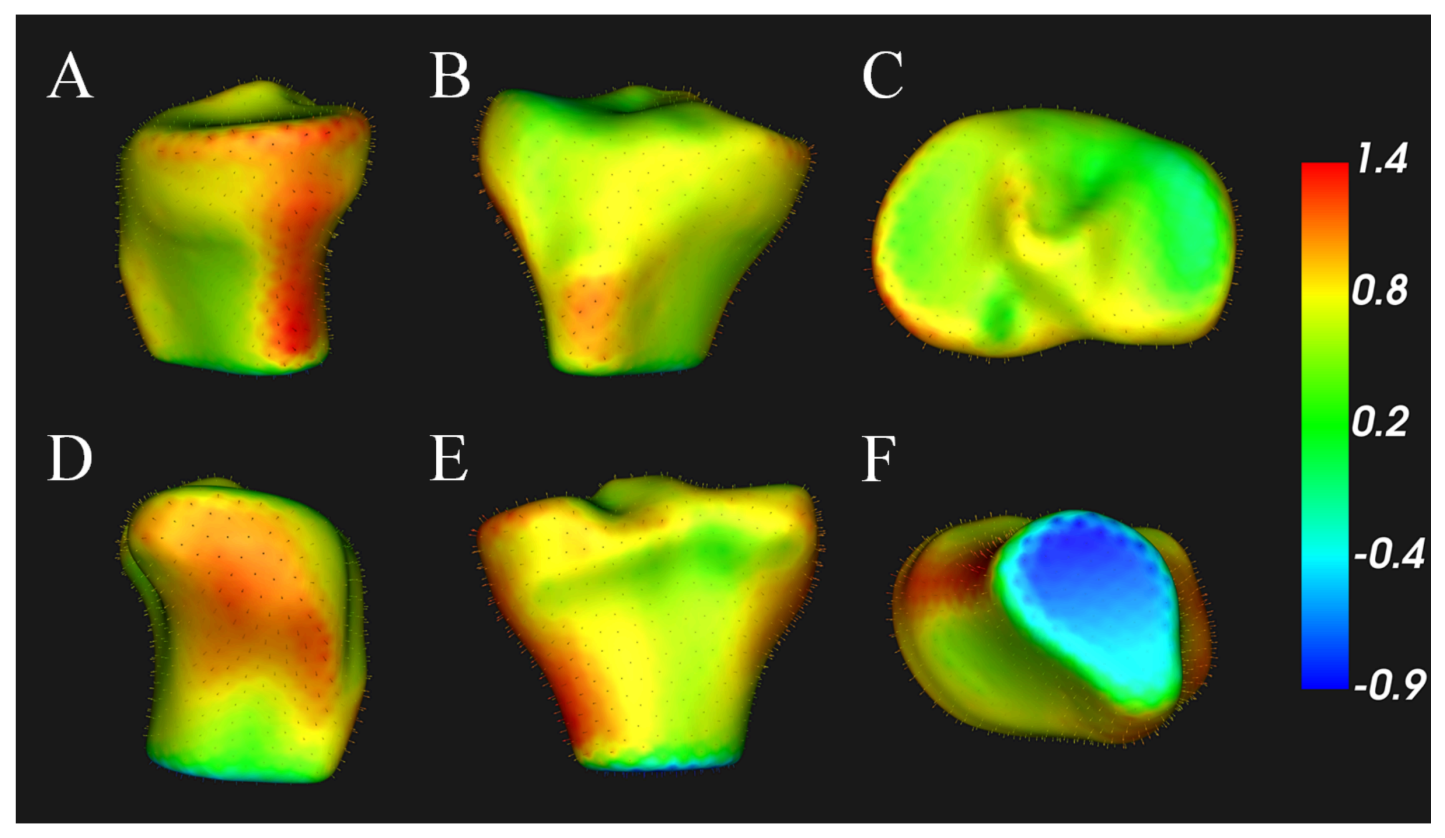
Descriptive surface map of between-group tibial shape differences. The map visualizes surface displacement between the GJH and HC group mean tibial shapes. (**A**) Left-side view; (**B**) anterior view; (**C**) superior view; (**D**) right-side view; (**E**) posterior view; (**F**) inferior view. The color bar indicates GJH−HC surface displacement (mm), with warm and cool colors representing positive and negative displacement, respectively. The more apparent displacement patterns were observed around the tibial tuberosity and surrounding anterior and peripheral proximal tibial contours, whereas displacement across the central tibial plateau appeared less pronounced. The map is descriptive, and the colors do not indicate pointwise statistical significance.

**Figure 4:**
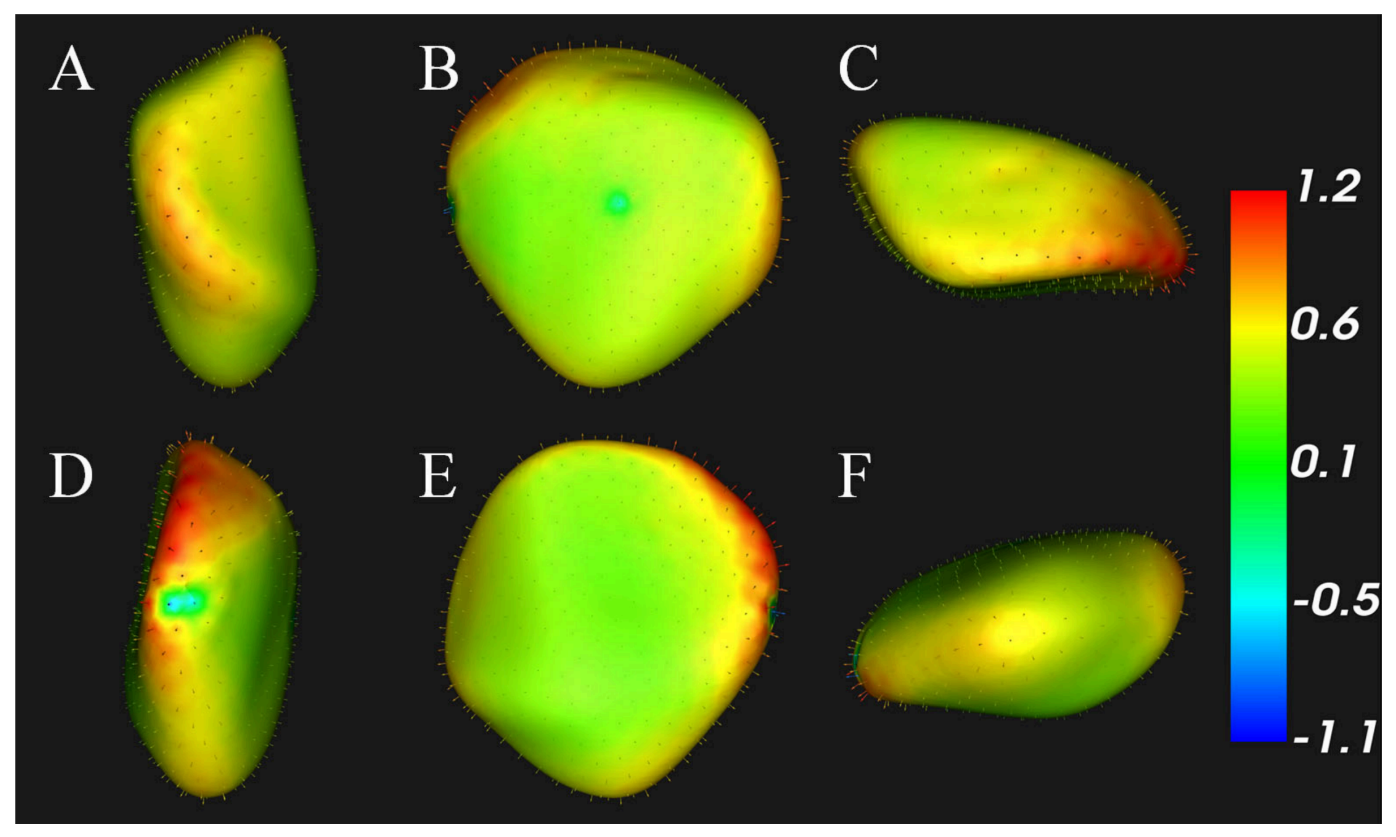
Descriptive surface map of between-group patellar shape differences. The map visualizes surface displacement between the GJH and HC group mean patellar shapes. (**A**) Left-side view; (**B**) anterior view; (**C**) superior view; (**D**) right-side view; (**E**) posterior view; (**F**) inferior view. The color bar indicates GJH−HC surface displacement (mm), with warm and cool colors representing positive and negative displacement, respectively. The more apparent displacement patterns were observed along the peripheral patellar margins, particularly the lateral and superolateral regions, whereas displacement across the central articular surface appeared less pronounced. The map is descriptive, and the colors do not indicate pointwise statistical significance.

These findings indicate that significant three-dimensional morphological differences remained between the GJH and HC groups after removal of global isotropic scale. Descriptive group-mean surface displacement maps for the femur, tibia, and patella are shown in Figure 2, Figure 3 and Figure 4, respectively.

### 3.3. Between-Group Differences in Individual PCs

**Figure 5:**
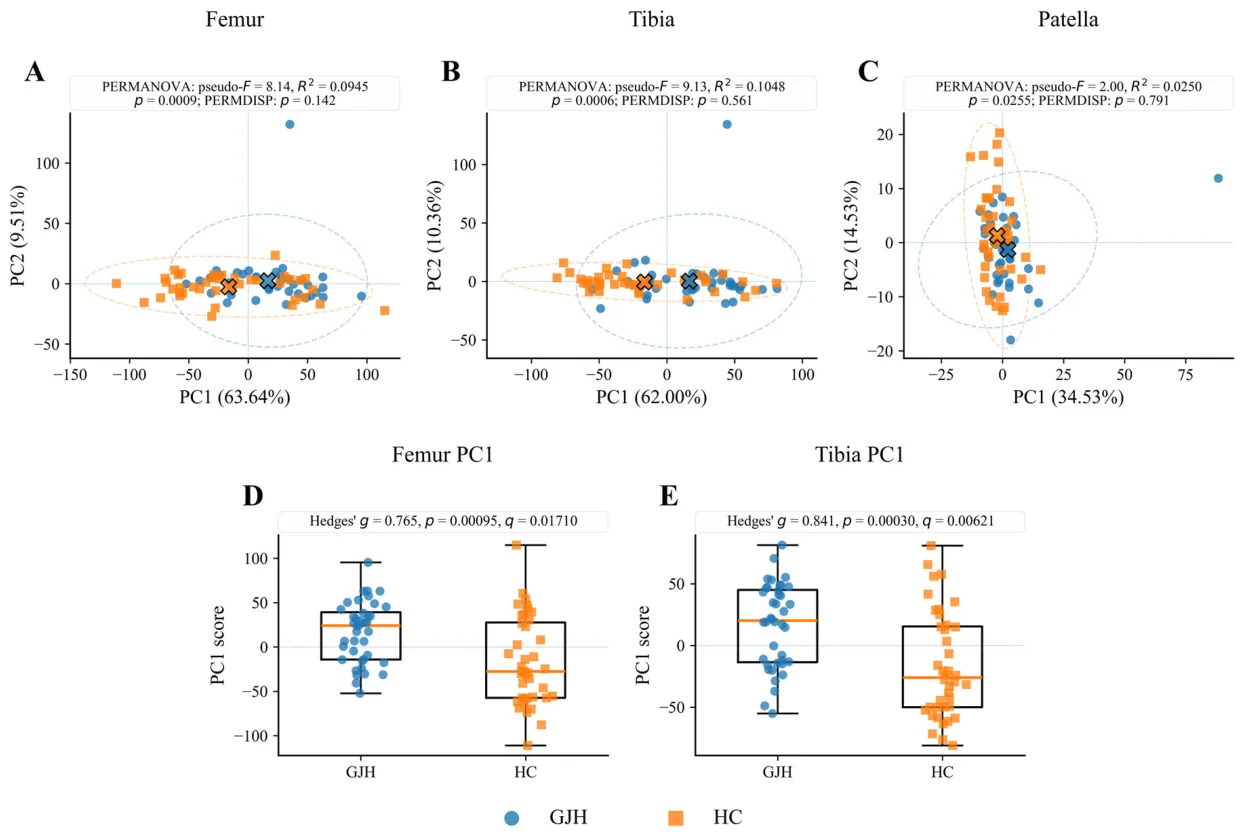
Statistical comparison of scale-normalized knee bone shape between the GJH and HC groups. (**A**–**C**) Two-dimensional visualization of PC1 and PC2 scores for the femur, tibia, and patella, respectively. Individual observations are shown together with group centroids and descriptive 95% covariance ellipses. Blue and orange “×” symbols indicate the group centroids for the GJH and HC groups, respectively. PERMANOVA was performed using Euclidean distances in the complete retained PC space, with 9999 permutations, and PERMDISP was used to assess homogeneity of multivariate dispersion. (**D**,**E**) Group distributions of femoral PC1 and tibial PC1 scores, respectively. Hedges’ *g*, unadjusted *p* values, and FDR-adjusted *q* values are reported. The multivariate statistical testing was based on all retained PCs accounting for at least 95% of cumulative variance.

**Figure 6:**
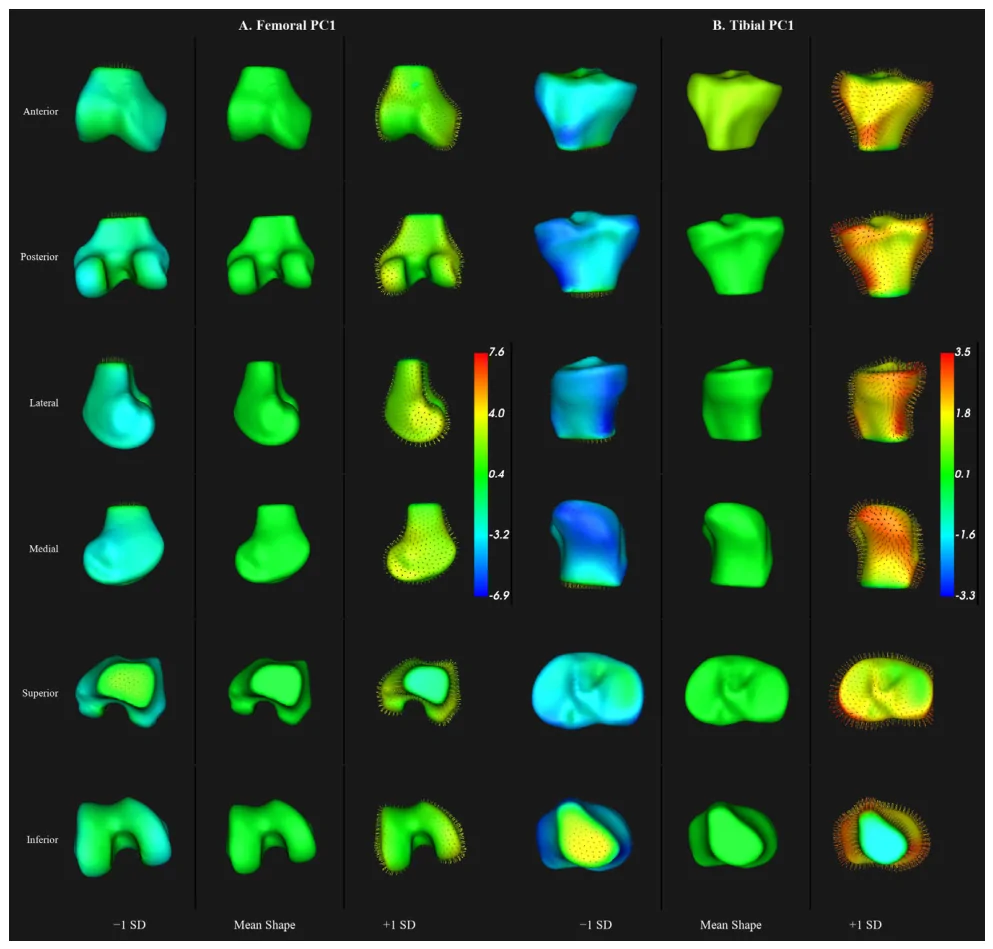
Anatomical visualization of the significant PC1 shape modes in the scale-normalized femoral and tibial statistical shape models. Reconstructed shapes at −1 SD, the population mean, and +1 SD along PC1 are shown for the femur (**A**) and tibia (**B**) in anterior, posterior, lateral, medial, superior, and inferior views. Surface colors represent displacement relative to the population mean shape, expressed in millimeters, and vectors indicate the direction of surface deformation. The sign of each principal component is arbitrary; therefore, −1 SD and +1 SD represent opposite directions along the same morphological mode. These visualizations illustrate the anatomical deformation represented by PC1 and do not indicate pointwise or regional statistical significance.

At the individual PC level, significant FDR-corrected between-group differences were observed for femoral PC1 and tibial PC1, whereas no individual patellar PC remained significant after FDR correction. Two-dimensional visualizations of the PC1–PC2 score distributions and the corresponding 95% covariance ellipses are shown in Figure 5A–C. Femoral PC1 differed significantly between groups, with higher scores in the GJH group than in the HC group (GJH: 16.43; HC: −16.43; difference = 32.85; Hedges’ g = 0.765; *p* = 0.00095; FDR-adjusted q = 0.0171). Tibial PC1 also differed significantly, with higher scores in the GJH group (GJH: 16.46; HC: −16.46; difference = 32.91; Hedges’ g = 0.841; *p* = 0.00030; FDR-adjusted q = 0.00621). The distributions of femoral PC1 and tibial PC1 scores are shown in Figure 5D,E, respectively. Reconstructed shapes at −1 SD, the population mean, and +1 SD along the significant femoral and tibial PC1 modes are shown in Figure 6. Descriptively, the femoral PC1 mode showed visible shape variation across the distal femoral condylar and periarticular contours, whereas the tibial PC1 mode showed visible variation across the proximal tibial plateau and adjacent proximal tibial contours. Patellar PC1 explained 34.53% of the total variance but did not differ significantly between groups after correction (Hedges’ g = 0.385, *p* = 0.0885, q = 0.561). Femoral PC16, tibial PC8, and patellar PC5 showed nominal between-group differences before multiple-testing correction but did not remain significant after FDR correction. Table 2 lists the explained variance, cumulative explained variance, GJH mean, HC mean, difference (GJH − HC), Hedges’ g, raw *p* value, and FDR-adjusted q value for each PC.

### 3.4. Contribution of Global Bone Size

In the scale-preserved analysis, PC1 accounted for 78.44% of femoral variance, 81.13% of tibial variance, and 76.73% of patellar variance. PC1 was strongly associated with segmented volume of the corresponding modeled region for the femur (r = −0.986, *p* < 0.001), tibia (r = −0.991, *p* < 0.001), and patella (r = 0.982, *p* < 0.001).

## 4. Discussion

This study used three-dimensional MRI and statistical shape modeling to compare the three-dimensional morphology of the femur, tibia, and patella between individuals with GJH and healthy controls. The main finding was that significant overall morphological differences remained in all three bones after removal of global isotropic scale. At the individual PC level, significant FDR-corrected differences were identified for femoral PC1 and tibial PC1, whereas the patella showed a significant overall multivariate difference without any individual PC remaining significant after FDR correction. Descriptive group-mean surface displacement maps showed visually larger displacements in several periarticular osseous contours than across the central articular surfaces. In the scale-preserved analysis, PC1 was strongly associated with segmented bone volume, indicating that global bone size contributed substantially to the dominant mode of variation before scale normalization. After global isotropic scale was removed, significant between-group differences remained for the femur, tibia, and patella, indicating that overall bone size alone did not account for the observed morphological differences.

On the descriptive femoral group-mean surface map, larger displacement patterns were visually apparent around the distal femoral periarticular contours, particularly in the epicondylar and paracondylar regions. Multi-view surface visualization suggested spatially distributed contour differences around the distal femoral periarticular margins. These regions provide an anatomical context for interpreting the observed femoral shape pattern. Anatomically, the medial epicondyle lies adjacent to attachment regions of the medial collateral ligament, medial patellofemoral ligament, posteromedial capsule, adductor magnus tendon, and medial head of the gastrocnemius [25]. The lateral epicondyle is adjacent to regions associated with the lateral collateral ligament, popliteus tendon, posterolateral complex, and lateral head of the gastrocnemius [26]. These anatomical relationships overlap with the regions where femoral contour differences were most apparent. The functional significance of these morphological differences may be further examined by integrating three-dimensional bone shapes with measures of soft-tissue function and knee stability.

On the descriptive tibial group-mean surface map, larger displacement patterns were visually apparent around the anterior tibial tuberosity and surrounding proximal periarticular contours. Differences across the central tibial plateau appeared less pronounced on the surface maps, with the more apparent variation occurring around peripheral and anterior proximal tibial regions. The tibial tuberosity is the attachment site of the patellar tendon and forms an important component of the knee extensor mechanism. Its anatomical relationship with the extensor mechanism provides relevant anatomical context for the contour differences observed in this region. The region surrounding the medial tibial condyle is associated with the pes anserinus, semimembranosus, medial collateral ligament, and medial joint capsule [25], while lateral proximal tibial regions are related to the iliotibial band, lateral joint capsule, and anterolateral stabilizing structures [26]. These periarticular anatomical relationships correspond to the regions where proximal tibial shape variation was most apparent. Pedoia et al. applied three-dimensional MRI-based SSM to knees with acute ACL injury and showed that bone shape analysis can identify injury-related structural characteristics [13]. This previous work demonstrates the utility of three-dimensional SSM for characterizing variations in proximal tibial morphology.

On the descriptive patellar group-mean surface map, larger displacement patterns were visually apparent along the peripheral margins, particularly in the lateral and superolateral regions. Unlike the femur and tibia, no individual patellar PC remained significant after FDR correction, indicating that the overall multivariate difference was not attributable to a single FDR-significant shape mode. The lateral and superolateral patellar margins are adjacent to the lateral retinaculum, joint capsule, vastus lateralis, and quadriceps tendon, providing anatomical context for the observed peripheral differences. Previous systematic review evidence has shown an association between joint hypermobility and patellar instability, suggesting that GJH may increase the risk of patellar dislocation or instability [7]. Fitzpatrick et al. showed that three-dimensional SSM can summarize variation in patellar and trochlear morphology, while Nagawa et al. used MRI-based statistical shape analysis to distinguish patellofemoral instability from normal femoral morphology and developed a machine-learning prediction model [14,15]. These studies support three-dimensional shape analysis of the patellofemoral joint. The present multivariate pattern suggests that the patellar group difference may be distributed across several dimensions of shape variation rather than represented by a single dominant morphological feature.

The potential biomechanical relevance of this study lies in the finding that three-dimensional differences in knee bone morphology remained detectable in individuals with GJH after removal of global isotropic scale. The descriptive spatial patterns involved several periarticular regions, including distal femoral epicondylar and paracondylar contours, the tibial tuberosity and surrounding proximal tibial contours, and peripheral patellar margins. Clouthier et al. showed that variations in knee geometry identified by SSM can affect knee kinematics, contact forces, and cartilage contact pressure [27]. Smoger et al. also demonstrated relationships between knee anatomy and kinematics [28]. These previous findings indicate that osseous geometry is relevant to knee biomechanics and provide a rationale for examining whether the between-group morphological differences observed here are associated with differences in joint motion or loading. The present findings therefore support future studies integrating SSM with dynamic kinematics, muscle function, and joint loading.

This study has several limitations. First, the cross-sectional design does not allow for determination of whether the observed bone morphological differences represent intrinsic structural characteristics of individuals with GJH, developmental adaptations, or consequences of long-term differences in joint loading and motor control. Second, this study assessed static morphology and did not directly measure dynamic knee motion, patellar tracking, or joint mechanics. Thus, the findings cannot establish that the observed morphological differences lead to abnormal kinematics or clinical symptoms. Third, Procrustes scaling removes global isotropic size differences but does not necessarily eliminate allometric relationships between size and shape; the scale-normalized results should therefore be interpreted as residual shape variation after removal of global scale. Fourth, the surface difference maps are descriptive and do not constitute pointwise or regional statistical tests. Future studies may integrate SSM with dynamic imaging, gait or landing analyses, cartilage imaging, joint space, and meniscal morphology to investigate the functional and structural relevance of GJH-related bone shape differences.

## 5. Conclusions

Using three-dimensional MRI and statistical shape modeling, this study found significant differences in femoral, tibial, and patellar morphology between individuals with GJH and healthy controls after removal of global isotropic scale. Femoral and tibial differences were primarily reflected in PC1, whereas the patella showed an overall multivariate difference without any individual PC remaining significant after FDR correction. Although global bone size contributed substantially to the dominant variation before scale normalization, the between-group morphological differences remained detectable after global isotropic scale was removed. These findings show statistically detectable differences in three-dimensional knee bone morphology between individuals with GJH and healthy controls and provide a rationale for future studies examining their relationships with knee stability and function.

## Figures and Tables

**Table 1:** Participant characteristics.

Variable	GJH (*n* = 40)	HC (*n* = 40)	*p* Value
Age (years)	20.63 ± 1.86	20.73 ± 1.95	0.815
Sex (male/female)	18/22	21/19	0.655
Height (cm)	168.47 ± 8.36	170.21 ± 9.76	0.392
Weight (kg)	63.12 ± 10.48	66.06 ± 14.71	0.306
BMI (kg/m^2^)	22.00 ± 2.86	22.56 ± 3.34	0.427
Beighton score	6.95 ± 1.11	1.30 ± 0.85	<0.001 *

Note: Continuous variables are presented as mean ± standard deviation. Sex was compared using the chi-square test, and all other variables were compared using independent-samples *t* tests. * Indicates a statistically significant between-group difference (*p* < 0.05). Abbreviations: GJH, generalized joint hypermobility; HC, healthy control; BMI, body mass index.

**Table 2:** Principal components retained in the scale-normalized SSMs to explain at least 95% of cumulative variance.

Bone	PC	Explained var.	Cum. var.	GJH Mean	HC Mean	Mean Diff. (GJH−HC)	Hedges’ g	*p* Value	FDR-Adjusted q Value
Femur	PC1	63.64%	63.64%	16.43	−16.43	32.85	0.765	0.00095	0.01710
	PC2	9.51%	73.15%	2.52	−2.52	5.04	0.286	0.20218	0.44874
	PC3	5.97%	79.12%	−1.83	1.83	−3.67	−0.262	0.24110	0.44874
	PC4	3.75%	82.87%	−0.40	0.40	−0.81	−0.072	0.74509	0.87818
	PC5	2.06%	84.93%	−1.34	1.34	−2.68	−0.328	0.14274	0.42821
	PC6	1.55%	86.48%	1.35	−1.35	2.70	0.381	0.08911	0.40099
	PC7	1.52%	88.00%	−1.21	1.21	−2.42	−0.345	0.12291	0.42821
	PC8	1.20%	89.20%	−0.36	0.36	−0.73	−0.115	0.60538	0.77834
	PC9	1.08%	90.28%	0.07	−0.07	0.13	0.022	0.92164	0.92164
	PC10	0.85%	91.13%	0.44	−0.44	0.89	0.168	0.45066	0.67599
	PC11	0.76%	91.89%	0.64	−0.64	1.28	0.257	0.24930	0.44874
	PC12	0.72%	92.60%	−0.44	0.44	−0.87	−0.179	0.42062	0.67599
	PC13	0.61%	93.21%	−0.62	0.62	−1.24	−0.279	0.21231	0.44874
	PC14	0.45%	93.66%	0.12	−0.12	0.24	0.062	0.78061	0.87818
	PC15	0.42%	94.08%	−0.05	0.05	−0.10	−0.027	0.90369	0.92164
	PC16	0.41%	94.49%	1.03	−1.03	2.07	0.579	0.01071	0.09635
	PC17	0.37%	94.87%	0.72	−0.72	1.44	0.418	0.06339	0.38037
	PC18	0.36%	95.22%	0.25	−0.25	0.51	0.147	0.50874	0.70440
Tibia	PC1	62.00%	62.00%	16.46	−16.46	32.91	0.841	0.00030	0.00621
	PC2	10.36%	72.36%	0.41	−0.41	0.82	0.047	0.83281	0.94582
	PC3	4.75%	77.11%	0.33	−0.33	0.66	0.056	0.80100	0.94582
	PC4	2.60%	79.71%	−0.13	0.13	−0.27	−0.031	0.89080	0.94582
	PC5	2.31%	82.03%	−0.60	0.60	−1.21	−0.147	0.50907	0.94582
	PC6	2.02%	84.05%	−0.86	0.86	−1.73	−0.226	0.31090	0.72543
	PC7	1.67%	85.73%	1.37	−1.37	2.74	0.399	0.07513	0.51200
	PC8	1.43%	87.16%	−1.43	1.43	−2.86	−0.454	0.04385	0.46038
	PC9	1.36%	88.52%	−0.75	0.75	−1.50	−0.239	0.28340	0.72543
	PC10	0.96%	89.48%	−0.97	0.97	−1.93	−0.371	0.09752	0.51200
	PC11	0.77%	90.25%	−0.62	0.62	−1.24	−0.264	0.23779	0.71337
	PC12	0.74%	90.99%	−0.04	0.04	−0.07	−0.015	0.94582	0.94582
	PC13	0.64%	91.63%	0.09	−0.09	0.17	0.040	0.85608	0.94582
	PC14	0.56%	92.20%	−0.13	0.13	−0.27	−0.065	0.76887	0.94582
	PC15	0.53%	92.72%	0.03	−0.03	0.07	0.017	0.93821	0.94582
	PC16	0.46%	93.19%	0.20	−0.20	0.41	0.112	0.61539	0.94582
	PC17	0.44%	93.62%	−0.53	0.53	−1.05	−0.297	0.18420	0.64468
	PC18	0.41%	94.04%	−0.52	0.52	−1.05	−0.306	0.17141	0.64468
	PC19	0.37%	94.40%	0.07	−0.07	0.14	0.042	0.85031	0.94582
	PC20	0.35%	94.75%	0.08	−0.08	0.17	0.052	0.81579	0.94582
	PC21	0.32%	95.08%	−0.28	0.28	−0.56	−0.183	0.41015	0.86131
Patella	PC1	34.53%	34.53%	2.19	−2.19	4.37	0.385	0.08853	0.56057
	PC2	14.53%	49.06%	−1.28	1.28	−2.55	−0.345	0.12388	0.56057
	PC3	9.77%	58.83%	−0.13	0.13	−0.25	−0.041	0.85256	0.95321
	PC4	7.16%	65.99%	0.59	−0.59	1.18	0.226	0.31137	0.79151
	PC5	5.60%	71.59%	1.02	−1.02	2.04	0.449	0.04666	0.56057
	PC6	4.14%	75.73%	0.64	−0.64	1.27	0.322	0.15066	0.56057
	PC7	3.51%	79.24%	−0.02	0.02	−0.05	−0.013	0.95321	0.95321
	PC8	2.61%	81.85%	−0.13	0.13	−0.25	−0.079	0.72353	0.95321
	PC9	2.14%	83.99%	0.31	−0.31	0.62	0.217	0.32980	0.79151
	PC10	1.61%	85.60%	0.52	−0.52	1.04	0.428	0.05717	0.56057
	PC11	1.36%	86.95%	−0.16	0.16	−0.32	−0.139	0.53157	0.91126
	PC12	1.26%	88.22%	−0.22	0.22	−0.44	−0.200	0.36972	0.80667
	PC13	1.06%	89.28%	−0.11	0.11	−0.22	−0.108	0.62664	0.95321
	PC14	1.00%	90.28%	0.04	−0.04	0.08	0.041	0.85403	0.95321
	PC15	0.79%	91.06%	−0.05	0.05	−0.10	−0.056	0.80022	0.95321
	PC16	0.70%	91.76%	−0.21	0.21	−0.42	−0.257	0.25037	0.75112
	PC17	0.58%	92.34%	0.01	−0.01	0.03	0.017	0.93862	0.95321
	PC18	0.54%	92.88%	0.12	−0.12	0.24	0.167	0.45367	0.83755
	PC19	0.49%	93.37%	−0.01	0.01	−0.02	−0.015	0.94567	0.95321
	PC20	0.46%	93.83%	−0.02	0.02	−0.03	−0.023	0.91875	0.95321
	PC21	0.38%	94.21%	0.20	−0.20	0.40	0.335	0.13392	0.56057
	PC22	0.36%	94.57%	0.04	−0.04	0.07	0.062	0.77913	0.95321
	PC23	0.34%	94.91%	0.18	−0.18	0.35	0.312	0.16350	0.56057
	PC24	0.32%	95.23%	−0.10	0.10	−0.20	−0.185	0.40550	0.81101

Note: Difference = GJH mean − HC mean. Raw *p* values are from Welch’s independent-samples *t* tests. FDR-adjusted q values were calculated using the Benjamini–Hochberg procedure within the retained PCs for each bone. Abbreviations: SSM, statistical shape modeling; PC, principal component; GJH, generalized joint hypermobility; HC, healthy control; FDR, false discovery rate.

## Data Availability

The datasets generated and analyzed during the current study are available from the corresponding author on reasonable request.

## Abbreviations

The following abbreviations are used in this manuscript:

ACL, anterior cruciate ligament; BMI, body mass index; DESS, dual-echo steady-state; FDR, false discovery rate; FOV, field of view; GJH, generalized joint hypermobility; HC, healthy control; MRI, magnetic resonance imaging; PC, principal component; PERMANOVA, permutational multivariate analysis of variance; SSM, statistical shape modeling; TE, echo time; TR, repetition time; ASSD, average symmetric surface distance; PCA, principal component analysis; PERMDISP, permutational analysis of multivariate dispersions.
